# William Henry Schieffelin

**Published:** 1895-08

**Authors:** 


William Henry Schieffelin, senior member of the long-established drug house of W. H. Schieffelin & Company, New York, died June 21, 1895, in the fifty-ninth year of his age. Mr. Schieffelin was one of the best known business men in the country and the house, of which he was the head, signalised the centennial anniversary of its establishment by issuing last year a handsome artistic brochure that was noticed in these columns. Mr. Schieffelin served creditably during the late war and was a companion of the Military Order of the Loyal Legion.

